# Stabilized Radial Basis Function Finite Difference Schemes with Mass Conservation for the Cahn–Hilliard Equation on Surfaces

**DOI:** 10.3390/e27121206

**Published:** 2025-11-28

**Authors:** Jinwei Qiao, Yuanyang Qiao, Yinnian He

**Affiliations:** 1 College of Mathematics and System Sciences, Xinjiang University, Urumqi 830046, China; 15525018659@163.com (J.Q.); qiaoyymath@126.com (Y.Q.); 2 School of Mathematics and Statistics, Xi’an Jiaotong University, Xi’an 710049, China

**Keywords:** Cahn–Hilliard equation, Laplace–Beltrami operator, RBF-FD method, mass conservation, projection method

## Abstract

It is well known that the Cahn–Hilliard equation satisfies the energy dissipation law and the mass conservation property. Recently, the radial basis function–finite difference (RBF–FD) approach and its numerous variants have garnered significant attention for the numerical solution of surface-related problems, owing to their intrinsic advantage in handling complex geometries. However, existing RBF–FD schemes generally fail to preserve mass conservation when solving the Cahn–Hilliard equation on smooth closed surfaces. In this paper, based on an $L^{2}$ projection method, two numerically efficient RBF–FD schemes are proposed to achieve mass-conservative numerical solutions, which are demonstrated to preserve the mass conservation law under relatively mild time-step constraints. Spatial discretization is performed using the RBF–FD method, while based on the convex splitting method and a linear stabilization technique, the first-order backward Euler formula (BDF1) and the second-order Crank–Nicolson (CN) scheme are employed for temporal integration. Extensive numerical experiments not only validate the performance of the proposed numerical schemes but also demonstrate their ability to utilize mild time steps for long-term phase-separation simulations.

## 1. Introduction

The Cahn–Hilliard (CH) equation is commonly utilized to depict phase separation phenomena in binary mixtures [1,2]. Its utility extends across various scientific domains, including spinodal decomposition of binary alloys [3], multi-phase fluid flows [4,5,6], image inpainting [7,8], thermally induced phase separation [9], modeling of martensitic phase transformation [10], grain growth [11], solidification and solid-state structural phase transformations [12,13,14], and tumor growth [15,16].

The CH equation is expressed in its split form as follows [17,18]:

(1) $$\begin{array}{c} \left\{\begin{array}{c} \frac{\partial u(x,t)}{\partial t}=\nabla \cdot \left[\nabla \mu \left(x,t\right)\right],\\ \mu \left(x,t\right)=-\epsilon^{2}\Delta u\left(x,t\right)+f\left(u\left(x,t\right)\right), \end{array}\right.\;x\in \Omega ,\;t>0, \end{array}$$

where u(x,t) is the real-valued unknown function that usually represents the phase variable, Ω is a two-dimensional or three-dimensional domain with the boundary ∂Ω,

(2) $$\begin{array}{c} f\left(u\right)=F^{\prime }\left(u\right)=u^{3}-u,\;F\left(u\right)=\frac{1}{4}{\left(u^{2}-1\right)}^{2} \end{array}$$

is the double-well potential function that represents free energy, and ϵ represents the thickness of the interfacial region separating the two phases. The CH equation preserves the mass of each component and the energy dissipative nature, i.e.,

(3) $$\begin{array}{c} M_{u}=\int_{\Omega }u_{0}\left(x\right)dx=\int_{\Omega }u\left(x,t\right)dx,\;\forall t>0 \end{array}$$

and

(4) $$\begin{array}{c} E\left(u\right)=\int_{\Omega }\left(F\left(u\right)+\frac{\varepsilon^{2}}{2}{\left|\nabla u\right|}^{2}\right)dx,\;\frac{d}{dt}E\left(t\right)\leq 0,\;\forall t>0. \end{array}$$

Therefore, the CH system exhibits two important physical properties: mass conservation and energy dissipation [18,19]. A recent development introduces a Lagrange multiplier strategy to incorporate additional physical constraints, such as mass conservation and bound preservation [20,21]. The cut-off technique [22], designed to ensure the maximal bound-preserving property, is also a type of projection strategy. From a theoretical perspective, considerable research has been conducted on the CH Equation (1). The physical background of the CH equation was investigated by C. M. Elliott [17], who also presented its numerical solution using the Galerkin finite element method. G. N. Wells [23] proposed a variational framework for the discontinuous Galerkin (DG) method, together with its numerical implementation and representative test cases. The resulting DG formulation eliminates the necessity of employing mixed finite element schemes, coupled systems, or highly continuous interpolation functions that were previously used to handle fourth-order spatial derivatives, as well as the finite difference method [24], spectral methods [25,26,27], etc.

The CH equation can be extended to a smooth closed surface Γ, and it also satisfies mass conservation and energy dissipation [28,29]. The CH equation on surface Γ adopts the following split form:

(5) $$\begin{array}{c} \left\{\begin{array}{c} \frac{\partial u(x,t)}{\partial t}=\Delta_{\Gamma }\mu \left(x,t\right),\\ \mu \left(x,t\right)=-\varepsilon^{2}\Delta_{\Gamma }u\left(x,t\right)+f\left(u\left(x,t\right)\right), \end{array}\right.\;x\in \Gamma ,\;t>0, \end{array}$$

where $\Delta_{\Gamma }$ is the Laplace operator on surface Γ.

In recent decades, numerous studies have been dedicated to developing efficient numerical schemes for the CH equation on surfaces aimed at preserving solution accuracy and these properties in discrete settings. In [30], Du et al. analyzed a finite element formulation for the CH equation defined on fixed surfaces and proved its convergence and well-posedness. The finite element method is well-suited for handling complex surface geometries that often arise in engineering applications. C. M. Elliott et al. [31] used the finite element method to solve the CH equation defined on an evolving surface driven by a prescribed velocity field. P. Gera et al. [32] analyzed the convergence properties of a closest-point formulation for the CH equation on surfaces. A local maximum entropy approximation was introduced by F. Amiri et al. [33] for the solution of the CH equation defined on smooth surfaces. Zhao et al. [34] examined two linearly stabilized, time-adaptive approaches for the CH equation on surfaces.

In recent years, a variety of mesh-free approaches have been developed to address partial differential equations (PDEs), including the global RBF collocation method [35] and localized RBF collocation method [36,37,38,39], etc. RBF–FD is a classical localized RBF collocation method. In contrast to mesh-based methods, which require structured meshes, the mesh-less feature significantly reduces the pre-processing time. RBF–FD methods operate on scattered nodes and provide greater flexibility in dealing with complex geometries. Moreover, they offer significant advantages when dealing with large-scale computational problems, discretizing differential operators on each local node stencil and reducing memory requirements. B. Fornberg et al. [40] presented some methods to solve PDEs with RBF, and highlight some recent results. Li et al. [41] developed a mesh-less local Petrov–Galerkin (MLPG) formulation using RBF collocation to investigate the convection–diffusion–reaction problem. High-order compact RBF–FD discretization for reaction–diffusion equations on smooth manifolds was presented by E. Lehto et al. [42]. Sun et al. [43] developed a mesh-free RBF scheme for solving the conservative Allen–Cahn equation defined on smooth compact surfaces, and validated the method’s accuracy and conservation capability through numerical tests on spherical and non-spherical geometries. The RBF–FD method, independent of spatial discretization, does not preserve mass conservation in surface Cahn–Hilliard equations. This study aims to investigate the use of the projection–correction mechanism within the numerical framework of the CH equation. In the present work, mass conservation is treated as an intrinsic physical constraint of (5). The intermediate solution is then projected onto a mass-conserving target set to enforce this constraint [44,45]. Moreover, we introduce a stabilizing term in the time-discrete scheme to preserve numerical stability during short time step sizes.

In this study, the main contributions can be summarized as follows:

- Two novel linear stabilized time-stepping BDF1 and CN schemes are constructed within an RBF–FD spatial discretization framework for the CH equation on surfaces.
- The mass conservation projection method is incorporated to optimize the obtained solution, to preserve the discrete mass conservation, and to satisfy the physical solution.
- Compared with BDF1 and CN schemes without a stabilizing term, the stability of the two schemes with stabilizing terms can still be preserved during a relatively short time-step constraint.

The structure of the paper is as follows. Section 2 outlines the spatial discretization methodology for the Laplace–Beltrami operator using the RBF–FD approach. Section 3 introduces the mass conservation projection method, employing an $L^{2}$ projection. Section 4 introduces the temporal discretization scheme, incorporating the mass-projection operator. Section 5 presents and discusses the numerical experiments in detail. Finally, Section 6 provides our concluding remarks.

## 2. Spatial Discretization

The following operators are expressed in Cartesian coordinates. This discussion focuses on two-dimensional surfaces embedded in $R^{3}$, due to their prevalence in practical applications. Let ∇ denote the standard gradient operator in $R^{3}$. Applying the standard gradient operator to a function *f* at a surface point x=(x,y,z), which is a closed smooth surface Γ, and projecting it onto the tangent space, yields the surface gradient $\nabla_{\Gamma }f$. Let $n={\left[n_{1},n_{2},n_{3}\right]}^{T}$ represent the unit normal vector on the surface Γ at point x. Then, ∇·n is the projection length of ∇ in the normal direction of the tangent plane at any point on the surface, and (∇·n)n is the projection vector of the gradient ∇ in the normal direction. Thus, the projection vector of the gradient ∇ on the tangent plane is ∇−(∇·n)n, which can be converted into ∇−n(n·∇). The surface gradient operator $\nabla_{\Gamma }$ and $\nabla_{\Gamma }f$ can take the following forms in Cartesian coordinates:

(6) $$\begin{array}{c} \nabla_{\Gamma }=\nabla -nn^{T}\nabla =\underset{P}{\underset{︸}{\left(I-nn^{T}\right)}}\nabla \end{array}$$

and

(7) $$\begin{array}{c} \nabla_{\Gamma }f=P\nabla f=\nabla f-nn^{T}\left(\nabla f\right), \end{array}$$

where I is a 3×3 identity matrix. Let P be the projection operator, which can be written in the following form:

(8) $$\begin{array}{c} P=\left[\begin{array}{ccc} p^{x} & p^{y} & p^{z} \end{array}\right]. \end{array}$$

Here, $p^{x}={\left[\left(1-n_{1}n_{1}\right),-n_{1}n_{2},-n_{1}n_{3}\right]}^{T}$, $p^{y}={\left[-n_{1}n_{2},\left(1-n_{2}n_{2}\right),-n_{2}n_{3}\right]}^{T}$, and $p^{z}={\left[-n_{1}n_{3},-n_{2}n_{3},\left(1-n_{3}n_{3}\right)\right]}^{T}$. By using this operator, the surface gradient $\nabla_{\Gamma }$ allows for the following expression:

(9) $$\nabla_{\Gamma }=P\nabla =\left[\begin{array}{c} p^{x}\cdot \nabla \\ p^{y}\cdot \nabla \\ p^{z}\cdot \nabla \end{array}\right]=\left[\begin{array}{c} Q^{x}\\ Q^{y}\\ Q^{z} \end{array}\right],$$

where $Q^{x}$, $Q^{y}$, and $Q^{z}$ denote the directional components. The surface Laplace operator, denoted by $\Delta_{\Gamma }$, is defined as the surface divergence of the surface gradient and is written in terms of $Q^{x}$, $Q^{y}$, and $Q^{z}$ as follows:

(10) $$\begin{array}{c} \Delta_{\Gamma }=\nabla_{\Gamma }\cdot \nabla_{\Gamma }=\left(P\nabla \right)\cdot \left(P\nabla \right)=Q^{x}Q^{x}+Q^{y}Q^{y}+Q^{z}Q^{z}. \end{array}$$

Consequently, the surface Laplace operator admits an explicit representation in Cartesian coordinates, which is subsequently employed in its numerical approximation.

Subsequently, we review the fundamentals of RBF interpolation. Consider a domain $\Omega \subseteq R^{d}$ and a kernel function ϕ:Ω×Ω→R that depends solely on the Euclidean distance between two points, i.e., ϕ(a,b)=ϕ(∥a−b∥) for a,b∈Ω. Let $X={\left\{x_{k}\right\}}_{k=1}^{N}$ be a discrete set of sample nodes on Ω, and denote it by *u*: Ω→R, a scalar function evaluated at these locations. The corresponding RBF interpolant is defined as follows:

(11) $$\begin{array}{c} I_{\phi }u\left(x\right)=\sum_{k=1}^{N}c_{k}\phi \left(∥x-x_{k}∥\right)+\sum_{l=1}^{q}\lambda_{l}p_{l}\left(x\right), \end{array}$$

where ${\left\{p_{l}\right\}}_{l=1}^{q}$ are polynomial basis functions and ${\left\{\lambda_{l}\right\}}_{l=1}^{q}$ are corresponding interpolation coefficients.

Various radial basis functions have been employed in practical computations, yet selecting the most suitable radial function for a specific problem remains unresolved. In the present work, we employ the multi-quadric (MQ) radial basis function, which is smooth and positive definite in $R^{d}$ and is commonly employed in RBF–FD schemes for the numerical solution of PDEs. Thus, we can construct RBF interpolation as follows:

(12) $$\begin{array}{c} I_{\phi }u\left(x\right)=\sum_{k=1}^{N}c_{k}\phi \left(∥x-x_{k}∥\right)+c_{N+1}, \end{array}$$

(13) $$\underset{A_{X}}{\underset{︸}{\left[\begin{array}{ccccc} \phi_{11} & \phi_{12} & \ldots & \phi_{1N} & 1\\ \phi_{21} & \phi_{22} & \ldots & \phi_{2N} & 1\\ \vdots & \vdots & \ddots & \vdots & \vdots \\ \phi_{N1} & \phi_{N2} & \ldots & \phi_{NN} & 1\\ 1 & 1 & \ldots & 1 & 0 \end{array}\right]}}\underset{c_{u}}{\underset{︸}{\left[\begin{array}{c} c_{1}\\ c_{2}\\ \vdots \\ c_{N}\\ c_{N+1} \end{array}\right]}}=\underset{u_{X}}{\underset{︸}{\left[\begin{array}{c} u_{1}\\ u_{2}\\ \vdots \\ u_{N}\\ 0 \end{array}\right]}},$$

where $\phi_{ij}=\phi \left(∥x_{i}-x_{j}∥\right)\;\;i,j=1,2,\ldots ,N$. We solve the interpolation coefficient ${\left\{c_{k}\right\}}_{k=1}^{N+1}$ by forming the above linear system (13) with the conditions ${\left.I_{\phi }u\right|}_{X}={\left.u\right|}_{X}$ and $\sum_{k=1}^{N}c_{k}=0$. The prerequisite for obtaining interpolation coefficients ${\left\{c_{k}\right\}}_{k=1}^{N+1}$ using $c_{u}=A_{X}^{-1}u_{X}$ is that the matrix $A_{X}$ is invertible. The invertibility of $A_{X}$ is ensured when ϕ is a positive definite radial function or satisfies the conditional positive definiteness.

Consider a discrete set of nodes $X={\left\{x_{k}\right\}}_{k=1}^{N}$ placed on the surface Γ. Assume that *u*: Γ→R is a differentiable function sampled on *X*. Select the (s−1) nodes $x_{2},\ldots ,x_{s}$ that are nearest to $x_{1}$ measured by the Euclidean distance in $R^{3}$. We refer to $x_{1}$ and its (s−1) nearest nodes as the stencil on the surface corresponding to $x_{1}$, denoted by $P_{1}={\left\{x_{j}\right\}}_{j=1}^{s}$. A local approximation of the operator $\Delta_{\Gamma }u$ at $x_{1}$ is then constructed as the weighted combination of the function values within the stencil $P_{1}$ as follows:

(14) $$\begin{array}{c} {\left.\left(\Delta_{\Gamma }u\right)\right|}_{x=x_{1}}\approx \sum_{j=1}^{s}w_{j}u\left(x_{j}\right). \end{array}$$

The first stencil $P_{1}$ is taken as an example, and the corresponding formulations for the other stencils can be derived in the same manner. The weights $w_{j}$ in this local approximation are obtained from the RBF interpolation system. As an initial step, an RBF interpolant for *u*, defined on the stencil $P_{1}$, is constructed as follows:

(15) $$\begin{array}{c} I_{\phi }u\left(x\right)=\sum_{j=1}^{s}c_{j}\phi \left(∥x-x_{j}∥)\right)+c_{s+1}. \end{array}$$

With the above derivation, the $Q^{x}$ component of the surface gradient under stencil $P_{1}$ is as follows:

(16) $${\left.\left(Q^{x}I_{\phi }u\left(x\right)\right)\right|}_{P_{1}}=\underset{K_{P1}^{x}}{\underset{︸}{\left[\begin{array}{ccccc} Q^{x}\phi_{11} & Q^{x}\phi_{12} & \ldots & Q^{x}\phi_{1s} & 0\\ Q^{x}\phi_{21} & Q^{x}\phi_{22} & \ldots & Q^{x}\phi_{22} & 0\\ \vdots & \vdots & \ddots & \vdots & \vdots \\ Q^{x}\phi_{s1} & Q^{x}\phi_{s2} & \ldots & Q^{x}\phi_{ss} & 0\\ 0 & 0 & \ldots & 0 & 0 \end{array}\right]}}\underset{c_{u}{|}_{P_{1}}}{\underset{︸}{\left[\begin{array}{c} c_{1}\\ c_{2}\\ \vdots \\ c_{s}\\ c_{s+1} \end{array}\right]}}.$$

Based on the preceding derivation, where $c_{u}{|}_{P_{1}}=A_{P_{1}}^{-1}u_{P_{1}}$, (16) can thus be reformulated as follows:

(17) $$\begin{array}{c} {\left.\left(Q^{x}I_{\phi }u\right)\right|}_{P_{1}}=K_{P_{1}}^{x}c_{u}=\left(K_{P_{1}}^{x}A_{P_{1}}^{-1}\right)u_{P_{1}}=Q_{P_{1}}^{x}u_{P_{1}}. \end{array}$$

Analogous approximations for the *y*- and *z*-components on this stencil can be derived as follows:

(18) $$\begin{array}{c} \begin{array}{cc} {\left.\left(Q^{y}I_{\phi }u\right)\right|}_{P_{1}} & =\left(K_{P_{1}}^{y}A_{P_{1}}^{-1}\right)u_{P_{1}}=Q_{P_{1}}^{y}u_{P_{1}}\\ {\left.\left(Q^{z}I_{\phi }u\right)\right|}_{P_{1}} & =\left(K_{P_{1}}^{z}A_{P_{1}}^{-1}\right)u_{P_{1}}=Q_{P_{1}}^{z}u_{P_{1}}. \end{array} \end{array}$$

Finally, a discrete analogue of the continuous surface Laplacian in (10) is constructed by substituting matrices $Q_{P_{1}}^{x}$, $Q_{P_{1}}^{y}$, and $Q_{P_{1}}^{z}$ for continuous operators $Q^{x}$, $Q^{y}$, and $Q^{z}$, yielding the discrete approximation of $\Delta_{\Gamma }u$ in stencil $P_{1}$ as follows:

(19) $$\begin{array}{c} {\left.\left(\Delta_{\Gamma }u\right)\right|}_{P_{1}}\approx \underset{L_{P_{1}}}{\underset{︸}{\left(Q_{P_{1}}^{x}Q_{P_{1}}^{x}+Q_{P_{1}}^{y}Q_{P_{1}}^{y}+Q_{P_{1}}^{z}Q_{P_{1}}^{z}\right)}}u_{P_{1}}, \end{array}$$

where $Q_{P_{1}}^{x}Q_{P_{1}}^{x}$, $Q_{P_{1}}^{y}Q_{P_{1}}^{y}$, and $Q_{P_{1}}^{z}Q_{P_{1}}^{z}$ all represent the multiplication of two matrices.

Although (19) provides approximations for all nodes within the stencil $P_{1}$, our primary interest lies in the approximation at the central node $x_{1}$. Owing to the nodes in $P_{1}$ being arranged in a fixed order, the RBF–FD weights $w_{j}$ in (14), are contained in the leading row of the matrix $L_{P_{1}}$. Extracting this row yields the desired weights for the node $x_{1}$. The same procedure is then applied to every node $x_{k}\in X$(k=1,…,N). Each node defines its own stencil $P_{k}$ by selecting its set of the nearest (s−1) nodes. For each stencil $P_{k}$, we can construct the matrix $L_{P_{k}}$ according to (19), and the RBF–FD weights associated with $x_{k}$ are obtained from the appropriate row of this matrix. Collecting all these weights forms a sparse N×N differentiation matrix *D*, which serves as the discrete approximation of the surface Laplacian.

## 3. Mass Conservation Projection Method

We first define the solution region on Γ, and variables are stored in

(20) $$\begin{array}{c} X=\left\{x_{k}|x_{k}=\left(x_{k},y_{k},z_{k}\right),k=1,\ldots ,N\right\}, \end{array}$$

where *N* represents the total node count. The discrete function space is as follows:

(21) $$\begin{array}{c} C=\left\{u:X\to R|u_{k}=u\left(x_{k},y_{k},z_{k}\right),k=1,\ldots ,N\right\}. \end{array}$$

Given a function v∈C, we define the discrete $L^{2}$ inner product as

(22) $$\begin{array}{c} {\langle u,v\rangle }_{e}=\sum_{m=1}^{d}S_{e_{m}}u_{m}v_{m}, \end{array}$$

and the associated discrete norm is provided by

(23) $$\begin{array}{c} {∥u∥}_{e}^{2}={\langle u,u\rangle }_{e}, \end{array}$$

where ${\left\{u_{m}\right\}}_{m=1}^{d}$ is the average value of u∈C, *d* is the total number of small surface blocks, and $S_{e_{m}}$ is the area of each small surface block. Given a function $u_{0}\in C$, $u_{0}$ represents a solution that has not been optimized through projection, and we project $u_{0}$ onto the admissible set as follows:

(24) $$\begin{array}{c} B=\left\{g\in C|{\langle g,1\rangle }_{e}={\langle u_{0},1\rangle }_{e}\right\}. \end{array}$$

Subsequently, the discrete $L^{2}$ projection is applied to enforce

(25) $$\begin{array}{c} u=\arg\min_{u\in C}\frac{1}{2}{∥u-u_{0}∥}_{e}^{2},\;{\langle u,1\rangle }_{e}={\langle u_{0},1\rangle }_{e}. \end{array}$$

For any $u,u_{0}\in C$, (25) can be reformulated into the following functional form by introducing a Lagrange multiplier ξ:

(26) $$\begin{array}{c} F\left(u,\xi \right)=\frac{1}{2}{∥u-u_{0}∥}_{e}^{2}+\xi {\langle u-u_{0},1\rangle }_{e}. \end{array}$$

We derive *u* from the minimization problem (26), which satisfies the following equation:

(27) $$\begin{array}{c} u_{m}=u_{0m}-\xi , \end{array}$$

where ${\left\{u_{0m}\right\}}_{m=1}^{d}$ is the average value of $u_{0}\in C$. Then, we can obtain ${\mathrm{mass}}_{\mathrm{initial}}={\langle u,1\rangle }_{e}=\sum_{m=1}^{d}S_{e_{m}}u_{m}=\sum_{m=1}^{d}S_{e_{m}}\left(u_{0m}-\xi \right)={\langle u_{0},1\rangle }_{e}$. Thus, the current mass is

(28) $$\begin{array}{c} {\mathrm{mass}}_{\mathrm{now}}=\sum_{m=1}^{d}S_{e_{m}}u_{0}m={\langle u_{0},1\rangle }_{e}+\xi \sum_{m=1}^{d}S_{e_{m}}={\langle u_{0},1\rangle }_{e}+\xi S, \end{array}$$

where *S* is the surface area. Therefore, we can obtain the Lagrange multiplier:

(29) $$\begin{array}{c} \xi =\frac{{\mathrm{mass}}_{\mathrm{now}}-{\langle u_{0},1\rangle }_{e}}{S}=\frac{{\mathrm{mass}}_{\mathrm{now}}-{\mathrm{mass}}_{\mathrm{initial}}}{S}. \end{array}$$

Once ξ is known, we can substitute ξ into $u_{m}=u_{0m}-\xi$ and obtain

(30) $$\begin{array}{c} u_{m}=u_{0m}-\frac{{\mathrm{mass}}_{\mathrm{now}}-{\mathrm{mass}}_{\mathrm{initial}}}{S}. \end{array}$$

At this stage, we have updated *u*, and we will denote the above process as $u=P_{h}u_{0}$ [44]. Hereafter, the mass conservation projection method is referred to as the MCP method.

**Lemma** **1** ([44])**.** *For any v∈C, it holds that $∥P_{h}{v∥}_{e}^{2}\leq {∥v∥}_{e}^{2}$.*

**Lemma** **2** ([44])**.** *For any v∈C and g∈B, it holds that $∥P_{h}{\left(v-g\right)∥}_{e}^{2}\leq {∥\left(v-g\right)∥}_{e}^{2}$.*

## 4. Temporal Integration

Let τ denote the uniform time step, $\tau =\frac{T}{n}$, *T* is the final time, and *n* is a positive integer. Then, define $t_{n}=n\tau$, $t^{n+1}=t^{n}+\tau$, $u^{n+1}=u\left(x,t^{n+1}\right)$, and $\mu^{n+1}=\mu \left(x,t^{n+1}\right)$.

### 4.1. The Linear Stabilized First-Order Backward Euler Formula (BDF1) Scheme

The operator $\Delta_{h}$ is introduced as the discrete counterpart of the surface Laplacian $\Delta_{\Gamma }$. Let ${\tilde{u}}^{n+1}$ and $u^{n+1}$ denote the preliminary and projected solutions at time $t_{n+1}$, respectively. The linear stabilized BDF1 scheme is formulated as follows:

(31) $$\begin{array}{c} \left\{\begin{array}{cc} {\tilde{u}}^{n+1} & =\tau \Delta_{h}{\tilde{\mu }}^{n+1}+u^{n}\\ {\tilde{\mu }}^{n+1} & =f\left(u^{n}\right)-\varepsilon^{2}\Delta_{h}{\tilde{u}}^{n+1}+\kappa \left({\tilde{u}}^{n+1}-u^{n}\right), \end{array}\right. \end{array}$$

where κ is a non-negative stabilizing parameter that needs to meet condition $\kappa \geq \underset{\rho \in [-1,1]}{max}\left|f^{\prime }\left(\rho \right)\right|$ [46]. Hereafter, the solution process of the above scheme can be written as the following linear system:

(32) $$\left[\begin{array}{cc} I & -\tau D\\ \varepsilon^{2}D-\kappa I & I \end{array}\right]\left[\begin{array}{c} {\tilde{U}}^{n+1}\\ {\tilde{V}}^{n+1} \end{array}\right]=\left[\begin{array}{c} U^{n}\\ F^{n}-\kappa U^{n} \end{array}\right]$$

where

(33) $$\begin{array}{cc} {\tilde{U}}^{n} & ={\left[\begin{array}{ccc} {\tilde{u}}_{1}^{n},{\tilde{u}}_{2}^{n}, & \ldots & ,{\tilde{u}}_{N}^{n} \end{array}\right]}^{T}\\ U^{n} & ={\left[\begin{array}{cccc} u_{1}^{n}, & u_{2}^{n}, & \ldots & ,u_{N}^{n} \end{array}\right]}^{T}\\ {\tilde{V}}^{n} & ={\left[\begin{array}{cccc} {\tilde{\mu }}_{1}^{n}, & {\tilde{\mu }}_{2}^{n}, & \ldots & ,{\tilde{\mu }}_{N}^{n} \end{array}\right]}^{T}\\ V^{n} & ={\left[\begin{array}{cccc} \mu_{1}^{n}, & \mu_{2}^{n}, & \ldots & ,\mu_{N}^{n} \end{array}\right]}^{T}\\ F^{n} & ={\left[\begin{array}{cccc} f(u_{1}^{n}), & f(u_{2}^{n}), & \ldots & ,f(u_{N}^{n}) \end{array}\right]}^{T} \end{array},$$

and *I* is an N×N identity matrix. Mass conservation can be guaranteed after the ${\tilde{u}}^{n+1}$ is updated using $u^{n+1}=P_{h}{\tilde{u}}^{n+1}$.

### 4.2. The Linear Stabilized Crank–Nicolson (CN) Scheme

Let ${\tilde{u}}^{n+\frac{1}{2}}$, ${\tilde{u}}^{n+1}$, and $u^{n+1}$ denote the intermediate, preliminary, and projected solutions at time $t_{n+1}$, respectively. We now formulate a linear, second-order CN scheme with stabilizing terms [46], which is expressed as follows:

(34) $$\left\{\begin{array}{c} {\tilde{u}}^{n+\frac{1}{2}}=\mathrm{BDF}1\left(u^{n},\tau /2\right),\\ \frac{{\tilde{u}}^{n+1}-u^{n}}{\tau }-\varepsilon^{2}\Delta_{\Gamma }\frac{{\tilde{u}}^{n+1}+u^{n}}{2}+f\left({\tilde{u}}^{n+\frac{1}{2}}\right)\\ \;+\kappa_{1}\left(\frac{{\tilde{u}}^{n+1}+u^{n}}{2}-{\tilde{u}}^{n+\frac{1}{2}}\right)+\kappa_{2}\tau \left({\tilde{u}}^{n+1}-u^{n}\right)=0. \end{array}\right.$$

The scheme can be understood as a two-step scheme. The first step is to use the BDF1 scheme to calculate ${\tilde{u}}^{n+\frac{1}{2}}$ as the prediction layer, and its scheme is as follows:

(35) $$\begin{array}{c} \mathrm{BDF}1:\left\{\begin{array}{cc} {\tilde{u}}^{n+\frac{1}{2}} & =\frac{\tau }{2}\Delta_{h}{\tilde{\mu }}^{n+\frac{1}{2}}+u^{n}\\ {\tilde{\mu }}^{n+\frac{1}{2}} & =f\left(u^{n}\right)-\varepsilon^{2}\Delta_{h}{\tilde{u}}^{n+\frac{1}{2}}+\kappa \left({\tilde{u}}^{n+\frac{1}{2}}-u^{n}\right) \end{array}\right.. \end{array}$$

It can be written as the following linear system:

(36) $$\left[\begin{array}{cc} I & -\frac{\tau }{2}D\\ \varepsilon^{2}D-\kappa_{1}I & I \end{array}\right]\left[\begin{array}{c} {\tilde{U}}^{n+\frac{1}{2}}\\ {\tilde{V}}^{n+\frac{1}{2}} \end{array}\right]=\left[\begin{array}{c} U^{n}\\ F^{n}-\kappa_{1}U^{n} \end{array}\right],$$

where the symbols are defined in the same way as (33). The second step applies the CN scheme to evaluate ${\tilde{u}}^{n+1}$ at the next time level, provided by

(37) $$\begin{array}{c} \mathrm{CN}:\left\{\begin{array}{cc} {\tilde{u}}^{n+1} & =\tau \Delta_{h}{\tilde{\mu }}^{n+1}+u^{n}\\ {\tilde{\mu }}^{n+1} & =f\left({\tilde{u}}^{n+\frac{1}{2}}\right)-\varepsilon^{2}\Delta_{h}\left(\frac{{\tilde{u}}^{n+1}+u^{n}}{2}\right)+\kappa_{1}\left(\frac{{\tilde{u}}^{n+1}+u^{n}}{2}-{\tilde{u}}^{n+\frac{1}{2}}\right)+\kappa_{2}\tau \left({\tilde{u}}^{n+1}-u^{n}\right), \end{array}\right. \end{array}$$

where $\kappa_{1}$ and $\kappa_{2}$ are two non-negative stabilizing parameters. The parameters are required to satisfy the conditions $\kappa_{1}\geq \underset{\rho \in [-1,1]}{max}\left|f^{\prime }\left(\rho \right)\right|$[46] and $\kappa_{2}\geq {\left(\frac{\kappa_{1}}{4}+\frac{\epsilon^{2}}{h^{2}}\right)}^{2}$[46]. Here, *h* denotes the planar mesh size, which can be approximated by the surface mesh size. Thus, the above scheme can be written as the following linear system:

(38) $$\left[\begin{array}{cc} I & \tau D\\ \frac{\varepsilon^{2}}{2}D-\left(\frac{\kappa_{1}}{2}+\tau \kappa_{2}\right)I & I \end{array}\right]\left[\begin{array}{c} {\tilde{U}}^{n+1}\\ {\tilde{V}}^{n+1} \end{array}\right]=\left[\begin{array}{c} U^{n}\\ F^{n+\frac{1}{2}}-\left[\frac{\varepsilon^{2}}{2}D-\left(\frac{\kappa_{1}}{2}+\tau \kappa_{2}\right)I\right]U^{n}-\frac{\kappa_{1}}{2}U^{n+\frac{1}{2}} \end{array}\right],$$

where the symbols are defined in the same way as (33). Still, using $u^{n+1}=P_{h}{\tilde{u}}^{n+1}$, $u^{n+1}$ is updated to ensure mass conservation. Finally, we can determine the *u* of each time layer to the final time in this way.

**Remark** **1.** *It follows from Lemmas 1 and 2 that the projection operator $P_{h}$ exhibits a contractive property under the discrete $L^{2}$ norm. Consequently, $P_{h}$ can serve as a corrective step that preserves both the discrete structure and mass conservation without degrading the overall accuracy of the underlying numerical scheme. The subsequent numerical experiments further confirm this observation.*

## 5. Numerical Experiments

This section presents a series of numerical experiments conducted to assess the performance of the proposed schemes, particularly the accuracy, convergence, and stability of the two linear stabilized schemes across various surfaces. The radial basis function chosen is multiquadric (MQ): $\phi \left(r\right)=\sqrt{c^{2}+r^{2}}$. The shape parameter *c* affects both the conditioning and interpolation accuracy of the resulting matrix. Generally, a larger *c* enhances the approximation accuracy but may cause a decrease in stability, while a smaller *c* improves stability at the cost of accuracy [47]. In all numerical examples below, the shape parameter is chosen as c=3, as it ensures favorable performance based on tests. Except for the numerical experiment presented in Section 5.3, all remaining experiments are conducted on the unit sphere. To achieve a balance between accuracy and stability, the stencil size is set to s=10 based on preliminary tests.

**Figure 1:**
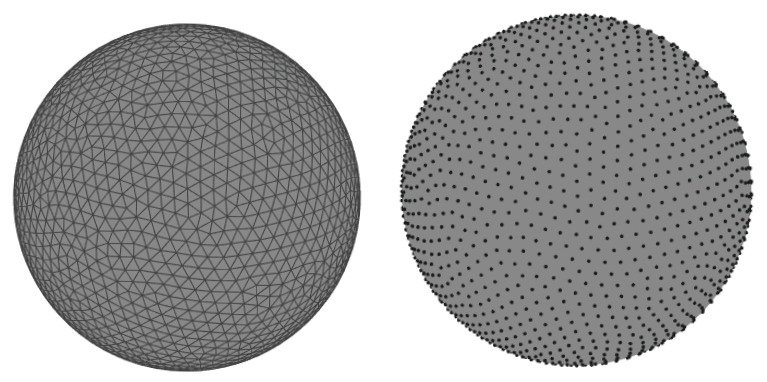
Uniform triangular mesh and point on a unit sphere.

**Figure 2:**
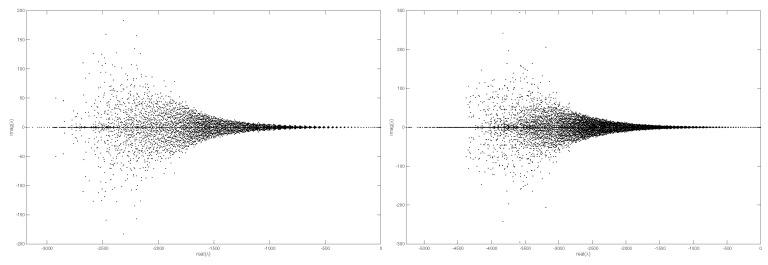
The eigenvalue distribution of the discrete weight matrix *D* of a unit sphere using N=4758 (**left**) and N=7584 (**right**).

Before we show the numerical experiments, we will first explain that all nodes *N* on the surfaces are each vertex that generates a uniform triangular mesh using Dismesh in MATLAB in Figure 1. Furthermore, Figure 2 shows the eigenvalue distribution of the discrete weight matrix *D* computed on the unit sphere, which indicates that all eigenvalues have negative real parts and confirms the stability of the discrete weight matrix *D*.

**Figure 3:**
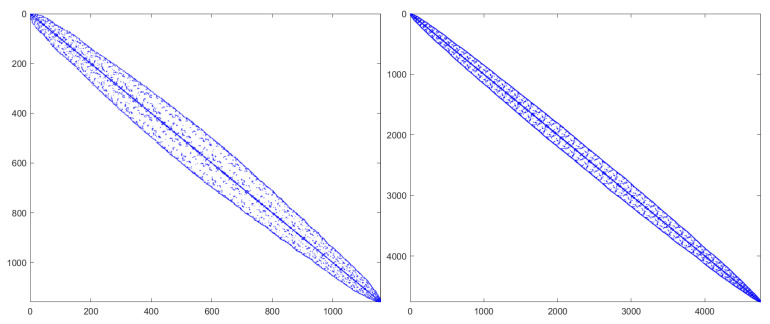
The re-ordered matrix *D* of a unit sphere using N=1158 (**left**) and N=4758 (**right**) using the RCM algorithm.

Figure 3 illustrates the sparsity pattern of the discrete weight matrix *D* using the reverse Cuthill–McKee (RCM) algorithm. The RCM algorithm is a sorting algorithm used to reduce matrix bandwidth and contour. It is widely used in fields such as finite element analysis and graph theory. It improves the efficiency of matrix operations by rearranging the rows and columns of the matrix to bring non-zero elements closer to the diagonal.

### 5.1. Convergence Tests

We evaluate the convergence of the BDF1 and CN schemes by solving the CH equation on the unit sphere. The discrete $L^{\infty }$ norm and $L^{2}$ norm are defined by $∥u-u_{h}{∥}_{\infty }=\underset{i\in [1,N]}{max}\left|u-u_{i}\right|$ and $∥u-u_{h}{∥}_{e}={\left(\sum_{m=1}^{d}S_{e_{m}}\left(u-u_{m}\right)\right)}^{\frac{1}{2}}$, respectively. For the stabilizing parameters, it was observed that any value within the above range defined in Section 4 has a negligible impact on the numerical results in our tests. Consequently, they are uniformly set to 2 in the following tests.

**Figure 4:**
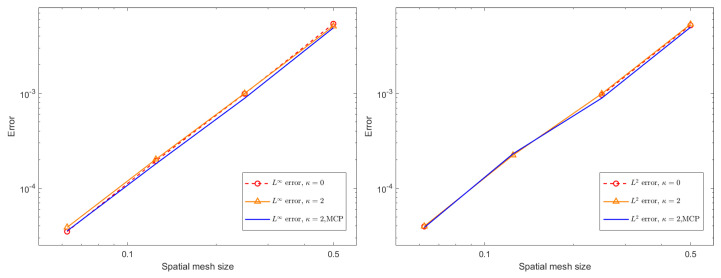
The spatial $L^{\infty }$ and $L^{2}$ errors for the BDF1 scheme with different stabilizing parameters, different mesh sizes *h*, and ϵ = 0.1.

**Figure 5:**
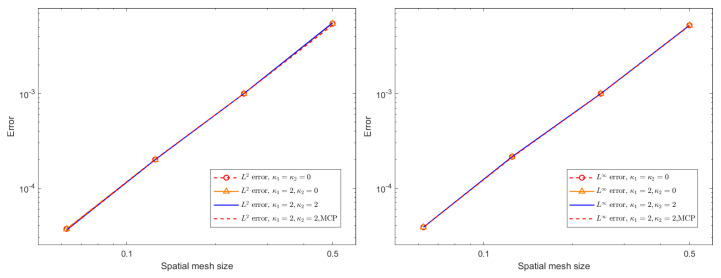
The spatial $L^{\infty }$ and $L^{2}$ errors for the CN scheme with different stabilizing parameters, different mesh sizes *h*, and ϵ = 0.1.

We study the following CH equation on the unit sphere $x^{2}+y^{2}+z^{2}=1$:

(39) $$\begin{array}{c} u_{t}=\Delta_{\Gamma }f\left(u\right)-\varepsilon^{2}\Delta_{\Gamma }^{2}u+G\left(x,t\right), \end{array}$$

where the source term G(x,t) is artificially constructed so that the exact solution is provided by $u\left(x,t\right)=0.1\left(t^{4}+1\right)\sin\left(x\right)\sin\left(y\right)$. Figure 4 and Figure 5 present spatial $L^{\infty }$ and $L^{2}$ errors, together with the corresponding convergence rates, for the BDF1 and CN time-stepping schemes under several choices of stabilization parameters. All computations are performed with a time step of $\tau =10^{-3}$ up to the final time, T=0.1. The results demonstrate that both schemes exhibit reduced error and clear algebraic convergence as the spatial resolution is refined. Furthermore, comparison across different stabilization strategies indicates that the added stabilization terms and mass conservation projection (MCP) do not deteriorate the accuracy of the method. Their influence on the measured errors is minimal, and the convergence behavior remains essentially unchanged. This confirms that the proposed stabilization framework is compatible with spatial accuracy and can be safely employed in large-scale simulations without compromising numerical fidelity.

**Figure 6:**
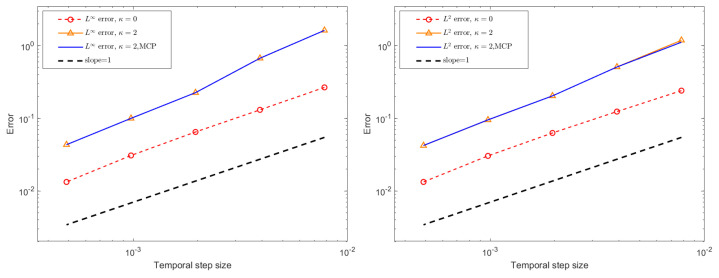
The temporal $L^{\infty }$ and $L^{2}$ errors for the BDF1 scheme with different stabilizing parameters, different τ, ϵ = 0.1, and h=0.05.

**Figure 7:**
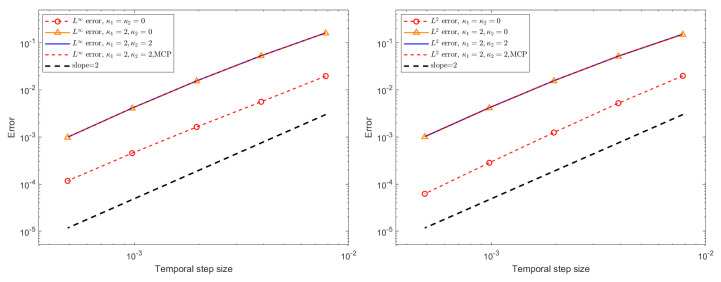
The temporal $L^{\infty }$ and $L^{2}$ errors for the CN scheme with different stabilizing parameters, different τ, ϵ = 0.1, and h=0.05.

For the assessment of temporal errors and convergence orders, the exact solution is not used. Instead, a highly resolved numerical solution computed with a very small time step, τ=1/8192, serves as the reference solution. Temporal $L^{\infty }$ and $L^{2}$ errors, together with the corresponding convergence rates for the BDF1 and CN schemes under different stabilization configurations, are reported in Figure 6 and Figure 7 for a final time of T=1. The numerical results confirm that the BDF1 scheme exhibits first-order accuracy in time, while the CN scheme achieves second-order accuracy, which is consistent with their theoretical design. Furthermore, although the stabilization terms and the mass conservation projection (MCP) do not alter the overall convergence behavior, their inclusion leads to a slight increase in the error magnitude. This modest accuracy deterioration is expected due to the dissipative nature of the stabilization terms, yet the impact remains minor and does not compromise the temporal convergence properties of the schemes.

**Figure 8:**
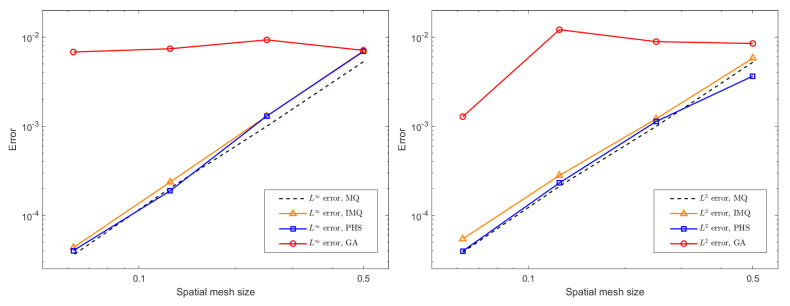
The spatial $L^{\infty }$ and $L^{2}$ errors for the CN scheme with different radial basis functions, different mesh sizes *h*, and ϵ = 0.1.

In this study, we adopt the MQ radial basis function tp construct the RBF–FD discretization. The MQ basis is widely used in mesh-free numerical methods due to its favorable approximation properties and its well-documented performance in solving partial differential equations on manifolds. Nevertheless, we acknowledge that other radial basis functions, including inverse multiquadrics (IMQ), Gaussians (GA), and polyharmonic splines (PHS), may exhibit different stability characteristics and accuracy levels. We conduct a brief spatial error test using the CN format, $\kappa_{1}=2$, $\kappa_{2}=2$, and MCP with the same parameter settings mentioned above. For the additional experiments involving alternative radial basis functions, the shape parameter of the IMQ basis was selected as 3, the shape parameter of the GA basis was set to 2, and the power parameter of the PHS basis was chosen as 2 based on preliminary testing. Figure 8 shows that the IMQ basis and PHS basis yield error magnitudes and convergence rates comparable to those obtained with the MQ basis, indicating that these alternatives provide similarly robust approximation properties within the RBF–FD framework. In contrast, the GA basis produces significantly larger errors and fails to achieve the expected convergence behavior, suggesting that its performance is less suitable for the present problem setting.

### 5.2. Stability Tests

This subsection examines the energy stability and mass conservation properties of the CH equation on the unit sphere. Two representative time-step sizes, $\tau =10^{-3}$ and τ=0.1, are considered. The initial condition is sampled from a random distribution in the interval [−0.9,0.9], with ε=0.1 and a node set of size N=4758.

**Figure 9:**
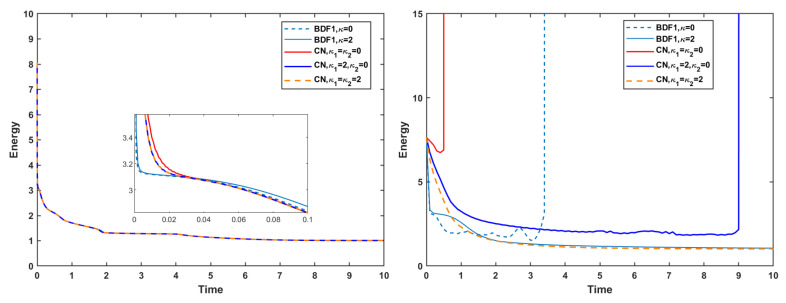
Evolutions of energy at time step $\tau =10^{-3}$ (**left**) and τ=0.1 (**right**) of the BDF1 and CN schemes with different stabilization terms.

Figure 9 illustrates the evolution of the discrete energy for various choices of stabilization terms. When the strict time step $\tau =10^{-3}$ is used, both the BDF1 and CN schemes exhibit monotone energy dissipation, and the energy remains numerically stable regardless of the stabilization terms. In contrast, at the relatively short time step τ=0.1, the behavior changes significantly: both schemes experience numerical blow-up when no stabilization term is applied or when only a single stabilization parameter is used. These observations confirm that stabilization terms play a crucial role in preventing numerical instabilities when larger time steps are employed.

**Figure 10:**
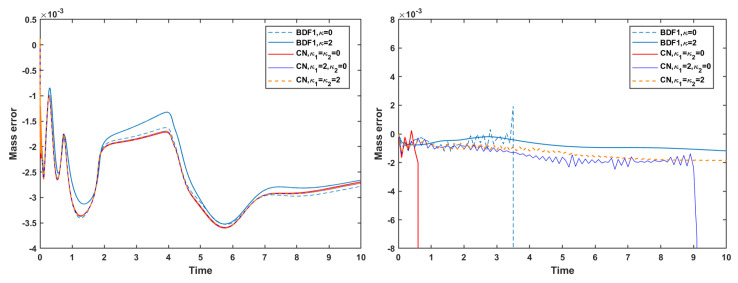
Mass error at time step $\tau =10^{-3}$ (**left**) and τ=0.1 (**right**) of the BDF1 and CN schemes with different stabilization terms.

Figure 10 reports the corresponding evolution of the mass error. Without the mass conservation projection (MCP), the mass error reaches the order of $10^{-3}$, indicating that neither scheme preserves mass to high accuracy, independent of the time-step size. Moreover, when stabilization is absent, both the BDF1 and CN schemes again exhibit numerical blow-up at the mild time step, reinforcing the conclusion that stabilization terms are essential for maintaining robustness in long-time or coarse-time-step simulations.

**Figure 11:**
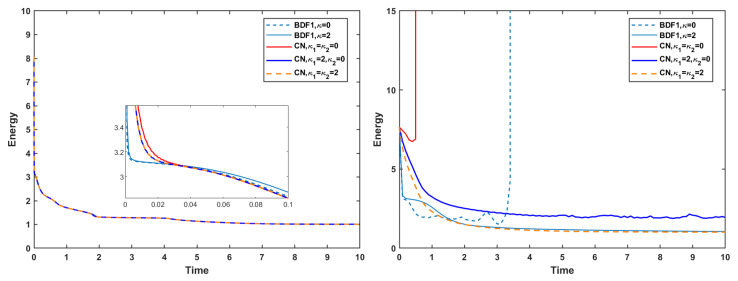
Evolutions of energy using the mass conservation projection method at time steps $\tau =10^{-3}$ (**left**) and τ=0.1 (**right**) of the BDF1 and CN schemes with different stabilization terms.

Thereafter, we incorporate the mass conservation projection (MCP) to further examine its impact on the evolution of the discrete energy and mass error. As shown in Figure 11, the use of MCP does not induce any substantial change in the energy evolution. The energy curves remain almost identical to those obtained without MCP, indicating that the projection procedure preserves the intrinsic energy-dissipation property of the underlying schemes.

**Figure 12:**
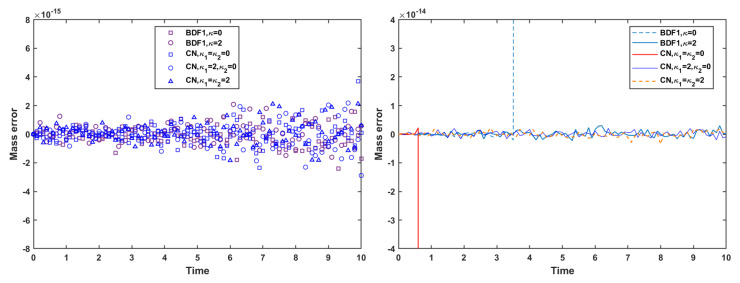
Mass error using the mass conservation projection method at time steps $\tau =10^{-3}$ (**left**) and τ=0.1 (**right**) of the BDF1 and CN schemes with different stabilization terms.

In contrast, the influence of MCP on mass is significant. Figure 12 shows that, with MCP applied, the mass error is reduced to the order of $10^{-15}$–$10^{-14}$ for both the strict time step and the mild time step. This confirms that the MCP effectively enforces mass conservation to near machine precision. However, it is also observed that when no stabilization terms are used, both the BDF1 and CN schemes still experience numerical blow-up at the short time step, even when MCP is applied. This highlights that MCP ensures accurate mass conservation but does not replace the need for stabilization in maintaining robustness at larger time steps.

### 5.3. Different Implicit Surfaces

**Figure 13:**
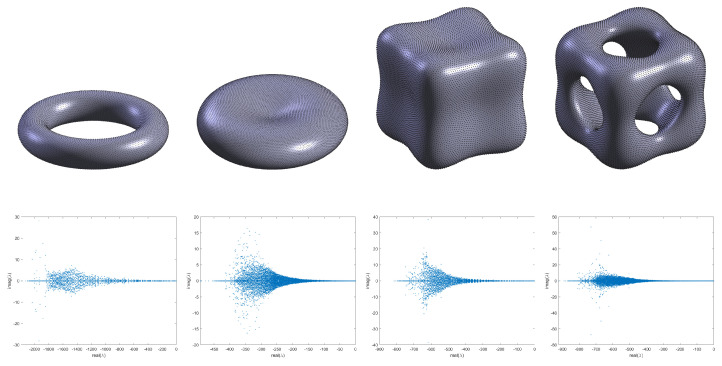
Example node sets for implicit surfaces and the eigenvalue distribution of the discrete weight matrix *D* of different surfaces (torus, red blood cell, tooth, and three cylindrical intersecting surfaces with 2224, 7584, 3482, and 8592 points).

**Figure 14:**
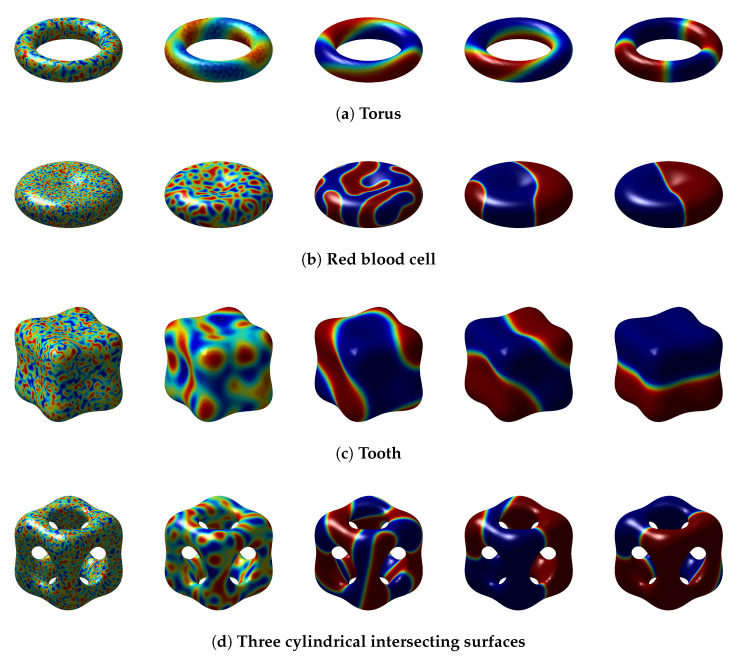
Numerical solutions on different surfaces.

Subsequently, we perform a series of numerical tests on the surface geometries illustrated in Figure 13. Furthermore, Figure 13 also shows the eigenvalue distribution of the discrete weight matrix *D* of different surfaces, which indicates that all eigenvalues have negative real parts and confirms the stability of these discrete weight matrices *D*. The simulations are conducted under the following unified parameter set: random initial values [−0.9,0.9], ϵ=0.1, $\tau =10^{-2}$, $\kappa_{1}=\kappa_{2}=2$, and s=7, with the MCP enabled throughout. The stencil size is set to s=7 because this can achieve ideal numerical simulation results. A larger stencil size can lead to instability and result in numerical explosion. As depicted in Figure 14, all surfaces exhibit clear phase separation phenomena during evolution. To further assess the robustness of the proposed numerical scheme across different geometries, we examine the corresponding energy evolution and mass conservation behavior on these four representative surfaces.

**Figure 15:**
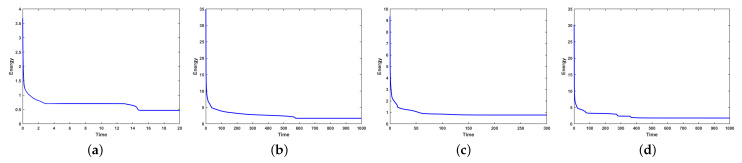
Evolutions of energy in the order of the above phase separation evolution on four surfaces ((**a**) torus, (**b**) RBC, (**c**) tooth, and (**d**) three cylindrical intersecting surfaces).

**Figure 16:**
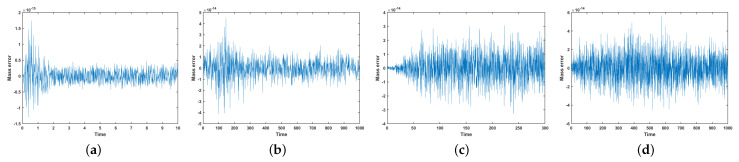
Mass error in the order of the above phase separation evolution on four surfaces ((**a**) torus, (**b**) RBC, (**c**) tooth, and (**d**) three cylindrical intersecting surfaces).

Figure 15 and Figure 16 show that the discrete energy decreases monotonically throughout the simulation for all surfaces, confirming that the scheme preserves the expected energy-dissipation property of the CH equation even in the presence of geometric complexity. In addition, the mass error remains near machine precision throughout the entire evolution, demonstrating that the scheme maintains excellent mass conservation across all tested geometries. These results demonstrate the robustness and broad applicability of the scheme in PDE computations on manifolds.

To further evaluate the computational performance of the proposed method, we present a detailed summary in Table 1, listing the CPU runtime required to simulate the phase separation process on the aforementioned surfaces in the experiment described in this section (all numerical experiments are performed on a workstation equipped with an Intel Core i5-12490F CPU (6 cores, 3.0 GHz) and 16 GB of RAM, running Windows 11 (64-bit). The algorithms are implemented in MATLAB R2025a.) The total CPU time corresponds to the full evolution until a steady state is achieved. This comparison highlights the efficiency of the algorithm under different numbers of nodes and geometries.

## 6. Conclusions

In this paper, we employ the RBF–FD approach for spatial discretization, coupled with the BDF1 and CN time-stepping schemes, to solve the CH equation on smooth closed surfaces. Incorporating the mass conservation projection method preserves the physical principle, while the inclusion of a time-stabilization term enhances stability, particularly for large time steps. The numerical schemes presented integrate the computational efficiency of the RBF–FD method with its capacity to fulfill physical solution and stability. Finally, a series of numerical experiments validates the theoretical findings and demonstrates the efficacy of the proposed scheme. While these results are promising, it is desirable to extend this approach to more complex surfaces [48,49].

## Figures and Tables

**Table 1:** CPU runtimes for simulating phase separation on various implicit surfaces.

Surface	*N*	Total CPU Time (s)
Torus	2224	337
Red blood cell	7584	29,101
Tooth	3842	5433
Three cylindrical intersecting surfaces	8592	28,741

## Data Availability

Data will be made available on request.
